# Lipid profiles and differential lipids in serum related to severity of community-acquired pneumonia: A pilot study

**DOI:** 10.1371/journal.pone.0245770

**Published:** 2021-03-11

**Authors:** Li Chen, Yali Zheng, Lili Zhao, Ying Zhang, Lu Yin, Yukun He, Xinqian Ma, Yu Xu, Zhancheng Gao

**Affiliations:** 1 Department of Respiratory & Critical Care Medicine, Peking University People’s Hospital, Beijing, China; 2 Department of Respiratory, Critical Care & Sleep Medicine, Xiang’an Hospital of Xiamen University, Xiamen, China; University of Pittsburgh, UNITED STATES

## Abstract

This study aimed to characterize the lipidomic responses to community-acquired pneumonia (CAP) and provide new insight into the underlying mechanisms of pathogenesis and potential avenues for diagnostic and therapeutic treatments. This study was performed from January 2017 to October 2018. Lipidomic profiles were generated using ultra high-performance liquid chromatography with tandem mass spectrometry (UHPLC-MS/MS) platform. Spearman’s rank correlation test and multiple linear regression analysis were applied to explore the correlation between changes in the relative abundance of lipids and clinical parameters. Kaplan–Meier methods were used to build 30-day survival curves. From the UHPLC-MS/MS results, a total of 509 and 195 lipid species were detected in the positive and negative ionization mode respectively. Positive ionization covered six lipid classes (glycerol-phospholipids, glycerolipids, sphingolipids, sterol-lipids, prenol-lipids, and fatty acid), whilst negative ionization covered three (glycerol-phospholipids, sphingolipids, fatty acid). Four lipids were selected as targets: PC (16:0_18:1), PC (18:2_20:4), PC (36:4), and PC (38:6). The relative increase of the areas under the curves for all four lipids were superior to the pneumonia severity index and CURB-65 (confusion, urea, respiratory rate, blood pressure, and age ≥65 years old) for discriminating severe CAP from CAP. Decreasing relative levels of PC (18:2_20:4), PC (38:6), and PC (36:4) were negatively related to fraction of inspiration O_2_; Changes in the relative abundance of PC (16:0_18:1) and PC (18:2_20:4) had significantly linear relationship with procalcitonin. Patients with an elevated level of PC (16:0_18:1) had significantly longer duration of hospital stays. As the relative abundance of PC (18:2_20:4), PC (36:4), and PC (38:6) decreased, the length of hospitalization days and 30-day mortality rate increased significantly (all log-rank *p*<0.05). Therefore, using the UHPLC-MS/MS platform’s serum lipidomic approach can help reveal changes in lipid abundance during CAP and establish lipid profiles related to disease severity.

## Introduction

Community-acquired pneumonia (CAP) remains a major health concern worldwide with substantial morbidity and mortality, especially among geriatric populations [1]. Moreover, the severity of clinical manifestations of CAP varies significantly [2, 3]. Owing to the diversity in clinical conditions and the lag in a clear definition of the causative pathogen, CAP remains a huge global challenge [4].

Lipids are the major components of alveolar surfactant [5]. In recent years, the rapid development of lipidomics has enabled us to gain new insights into the mechanism of disease development [6, 7]. Lipids play a critical role in cellular energy storage, structure, and signaling [8]. They are not only considered as the components of membranes but also act as an indispensable factor in the immune response by organizing signaling complexes in cellular membranes [9]. Studies have confirmed that lipids act as important inflammatory mediators during the infection process [7, 9, 10]. Changes in lipid components of the serum or plasma can occur during acute lung injury, sepsis, bacteremia, and viral infections [11–14]. Bioactive lipids located further downstream in the biological system can better reflect the body’s redox balance, oxidative stress, signaling, apoptosis, and inflammation than biological and gene expression biomarkers in body fluids; therefore, lipids provide more relevant and richer features in CAP. However, none of the studies thus far have focused on changes in lipid abundance of the serum of patients with different degrees of CAP.

In the current study, untargeted lipidomic analyses using high-performance liquid chromatography-mass spectrometry (HPLC-MS) were performed to identify CAP-related lipidomic signatures. The relationship between the abundance of identified lipids and the severity of CAP was then investigated. The characterization of the lipidomic response in CAP patients can help to further understand the heterogeneity of the disease and provide new insights for diagnosis and treatment.

## Materials and methods

### Study population

This study was performed from January 2017 to October 2018 and included patients hospitalized at the Peking University People’s Hospital (PKUPH), Fujian Provincial Hospital, Sichuan University West China Hospital. This study was registered at ClinicalTrials.gov (NCT03093220) and was endorsed by the Institutional Review Board of the PKUPH. All CAP patients were recruited from the Respiratory Medicine Department or Intensive Care Unit. All participants provided written informed consent prior to the collection of any data. Authors had access to information that could identify individual participants during and after data collection. The study was approved by the Medical Ethics Committee of the PKUPH (No. 2016PHB202-01).

Inclusion criteria for the study were evidence of pulmonary infiltrate on the chest, a chest radiograph showing either a new patchy infiltrate, leaf or segment consolidation, ground-glass opacity, or interstitial change [4, 15]. Additionally, the inclusion criteria included at least one of the following signs: (a) the presence of cough, purulent sputum production, and dyspnea; (b) fever (core body temperature >38.0°C); (c) auscultatory findings of abnormal breath sounds and rales; or (d) leukocytosis or leukopenia (peripheral white blood cell counts >10 × 10^9^/L or <4 × 10^9^/L), and if symptom onset began in communities. Severe CAP (SCAP) was defined as the presence of at least one major criterion or at least three minor criteria published by the American Thoracic Society in 2007 [2]. The major criteria were: invasive mechanical ventilation and septic shock with the need for vasopressors; the minor criteria included respiratory rate ≥30 breaths/minute, oxygenation index ≤ 250, multipolar infiltrates, confusion or disorientation, uremia (BUN level ≥ 20 mg/dL), leukopenia (WBC count <4000 cells/mm^3^), thrombocytopenia (platelet count <100,000 cells/mm^3^), hypothermia (core temperature <36°C), and hypotension requiring aggressive fluid resuscitation.

The exclusion criteria were age <18 years or the presence of any of the following: women who were pregnant or lactating, patients who had surgery within 3 months before onset, patients with evidence of nosocomial infections, and immunosuppressive conditions. Furthermore, patients with malignant tumor, chronic neurological diseases (e.g., Parkinson’s disease, multiple system atrophy), end-stage renal or liver disease, and active tuberculosis or pulmonary cystic fibrosis were excluded as well.

Demographic information was obtained using standard forms (including age, gender, smoking history, underlying diseases, complications, symptoms, signs, laboratory test results, and clinical treatment methods). Patient outcomes were evaluated at discharge and assessed through structured telephone interviews 30 days after enrolment (S1 File).

### Blood sample collection and preparation

A total of 5 mL of fasting peripheral venous blood was drawn from adult CAP patients during the first 72 h of hospital admission, injected into the sterile pro-coagulation tubes, and allowed to stand at room temperature for 30 min. The sample was centrifuged and the serum was aliquoted into 2–3 Eppendorf tubes and stored at -80°C for processing.

At the time of the assay, the serum samples were thawed on ice and centrifuged at 14,000 g at 4°C for 20 min, and then 100 μL of the supernatant was transferred to a new Eppendorf tube. Next, 400 μL of chloroform/methanol (v/v = 2:1) was added to the supernatant liquid and the tube was vortexed for 1 min and left to stand for 20 min; this process was repeated thrice after which the tube was centrifuged at 12,000 rcf for 5 min. The lower chloroform layer was transferred using a glass syringe and dried under nitrogen. Finally, the lipid samples were stored at -80°C in the freezer as dry pellets. To monitor the repeatability and stability of the analytical system, a pooled quality control (QC) sample solution was prepared and extracted, by combining 10 μL aliquots of each sample using the process described above.

Lysophosphatidylethanolamine (LPE) (14:0), phosphatidylethanolamine (PE) (14:0_14:0), phosphatidylcholine (PC) (14:0_14:0), phosphatidylserine (PS) (14:0_14:0), and D5-triglyceride were used as internal standards in the analyses. The average coefficient of variation (CV) of the internal standards was 21% in all the samples and 10.7% in the QC samples; which is in the acceptable range for the analysis.

### Untargeted UHPLC-MS/MS method for lipid analysis and lipid identification

Reverse-phase chromatography was selected for the LC separation using Cortecs C18 column (2.1×100 mm, Waters). Mobile phase A was prepared by dissolving 0.77 g of ammonium acetate in 400 mL of HPLC-grade water, followed by the addition of 600 m of HPLC-grade acetonitrile (PH ~7). Mobile phase B was prepared by mixing 100 mL of acetonitrile with 900 mL of isopropanol. The UHPLC system Ultimate 3000 (Thermo Fisher, CA) coupled with Q Exactive (Orbitrap) mass spectrometer (Thermo Fisher, CA) was used to acquire raw data. Details of parameters of the Q Exactive mass spectrometer and HPLC conditions are described in S2 File.

LipidSearch software v4.1.16 (Thermo, CA) was used to identify and quantify lipids containing more than 1,500,000 theoretical MS/MS fragment ions from 18 major lipid classes in the database, based on accurate precursor mass and characteristic fragments [16]. Lipid identification was based on a MS/MS match. The mass tolerance for the precursor and fragment was 8 ppm and 10 ppm, respectively. Only lipids with a chromatographic area >5E6 were regarded as a confident identification. A retention time shift of 0.25 min was allowed for quantitation. A retention time shift of 0.15 min was performed for “alignment.” M-score and chromatographic areas were used to reduce false positives. The criteria to eliminate the false positives were lipid species dependent. In addition, since ammonium acetate was used in the mobile phase, the adducts of + H, + NH_4_ were used in the positive mode search, and—H, + CH_3_COO in the negative mode [17]. Finally, mass spectrum data composed of chemical formula, detected the ion adduct with accurate precursor mass, retention time, peak shape and distribution of integrated peaks, and MS/MS fragment match were obtained (S3 File).

### Analysis and processing of UHPLC-MS/MS lipidomics data

It was necessary to pre-process the data to reduce the interference in the information and facilitate the mining of more meaningful biological information. The data pre-processing was performed using MetaboAnalyst 4.0 [18]. To remove the noise and improve the data quality, a lipid was included in the subsequent data analysis if it had a non-zero value for at least 80% of the samples of any group. Missing value estimations and filling in the data matrix were performed. Considering that most of the missing values were caused by low-abundance compounds (i.e., those below the detection threshold), we replaced all the missing values with small values (the half of the minimum positive values in the original data). We normalized the original abundance matrix of lipids to correct for the effects of factors such as individual differences or differences in sample collection or processing on absolute compound concentrations using constant sum, log transformed and auto-scaling (mean-centered and divided by the standard deviation of each variable) methods. The obtained normalized data were further analyzed.

#### Multidimensional statistical analysis

The normalized data were imported into SIMCA-P 14.0 (Umetrics, Sweden) for multivariate variable analyses (MVA). First, a principal component analysis (PCA) model was established to observe the overall distribution of samples in each group, explore possible factors affecting sample aggregation, and identify outliers. Second, according to the clinical grouping, an orthogonal partial least squares discriminant analysis (OPLS-DA) model was established for the samples to extract the different information between the groups, and the variable importance on projection (VIP) value was obtained. A VIP value greater than 1 indicated that the between-group differences were greater than the within-group differences. We used the R2X, R2Y, and Q2 parameter values to determine the quality of the OPLS-DA model. The quality of the MVA models was evaluated using a cross-validation analysis of variance (CV-ANOVA) and permutation test (500 iterations) [19].

### Statistical analyses

Categorical variables are expressed as numbers (percentages) and analyzed using a chi-square test or Fisher’s exact test. The Kolmogorov–Smirnov test was used to evaluate the distribution of continuous variable data. Normally distributed continuous variables were expressed as means ± standard deviations (mean ± SD) analyzed using the Student’s t-test or analysis of variance with post-hoc Tukey HSD test. Continuous nonparametric data were presented as medians and interquartile ranges (25th and 75th percentiles) and analyzed using the Mann–Whitney U or Kruskal–Wallis H test, as appropriate. A heat-map with a Euclidean distance measure of relative intensity of metabolites (logarithmic scale) and a Pearson’s correlation heatmap were generated using MetaboAnalyst 4.0 (Wishart Research Group, University of Alberta, USA) [18]. The receiver operating characteristic (ROC) curve was analyzed using the multivariate logistic regression data, and the area under the curve (AUC), sensitivity, specificity and 95% confidence interval (95% CI) were calculated to evaluate the performance of lipids and clinical indicators. The Youden index (farthest to diagonal line) was used to determine an optimal cut-off point for test results (Classification: include cut-off value for positive classification; Test direction: larger test result indicates more positive test). The two-tailed Spearman’s rho test correlation coefficient (r) was calculated to evaluate the strength and direction of the linear relationship between the abundance of target lipids and clinical indicators. Furthermore, to adjust the multicollinearity inherent in lipidomics data, a stepwise approach was used to perform multiple linear regression (MLR) analysis. The Kaplan–Meier method was applied to establish a 30-day survival curve, and logarithmic rank tests were utilized to compare the survival rates. Cox proportional hazards regression analysis was used to analyze the effect of target lipids on 30-day survival.

Statistical tests were performed using the SPSS statistics version 19.0 (IBM, NY, USA) and MedCalc Software version 15.8 (MedCalc Software, Ostend, Belgium).

## Results

### Demographic and clinical characteristics of participants

The final study population consisted of 28 patients with CAP (15 non-severe CAP [NSCAP] and 13 severe CAP [SCAP]) and 20 age, sex, and underlying disease matched non-CAP controls (NC). As indicated in Table 1, there were a range of etiologies of CAP, including bacteria, viruses, and fungi, which was typical for a heterogeneous CAP patient population. In particular, there were no significant differences in age, sex, smoking history, smoking index, and underlying disease among the three groups (*p* > 0.05). However, in the laboratory tests, the inflammatory response-related indicators such as the percentages of neutrophil (NE%), lymphocyte (LY%), and monocyte (MO%) and the levels of white blood cell (WBC), neutrophil (NE), lymphocyte (LY) and monocyte (MO) were all significantly different in the SCAP group compared with that in the NSCAP group (all *p* < 0.05). The levels of serum C-reactive protein (CRP) and procalcitonin (PCT) were both higher in the SCAP group (*p* < 0.05). In terms of physical examination, respiratory frequency of SCAP patients dramatically increased (*p* < 0.05). CURB-65 (confusion, urea, respiratory rate, blood pressure, and age ≥65 years) and pneumonia severity index (PSI) were prominently higher in the SCAP group than in the NSCAP group (*p* < 0.05). Comparing the detection of pathogens between the two groups, we found that the detection rate of bacteria in patients with SCAP was higher. In addition, hospitalization days and 30-day mortality were both substantially higher in patients with SCAP than in those with NSCAP (*p* < 0.05) (Table 1).

**Table 1:** Demographic and clinical characteristics of 48 study participants.

Characteristic	SCAP	NSCAP	NC	*p* value
	(N = 13)	(N = 15)	(N = 20)	
Male sex—no. (%)	8 (61.5)	6(40.00)	7(35.0)	0.304^a^
Age—years	62.08±18.21	65.40±17.86	60(48.25–67)	0.141^c^
Smoking history—no. (%)	4(30.80)	5(33.30)	3(15.00)	0.396^a^
Smoking index	0(0–14.50)	0(0–10.00)	0(0–0)	0.533^a^
**Underlying diseases**—no. (%)				
COPD	0 (0)	1 (6.70)	0 (0)	0.325^a^
Asthma	2 (15.40)	1 (6.70)	0 (0)	0.203^a^
Bronchiectasis	0 (0)	1 (6.70)	0 (0)	0.325^a^
Interstitial lung Disease	1 (7.70)	0 (0)	0 (0)	0.253^a^
Hypertension	4 (30.80)	5 (33.30)	5 (25)	0.856^a^
Cardiovascular disease	3 (23.10)	0 (0)	4 (20)	0.151^a^
Cerebrovascular disease	1 (7.70)	0 (0)	2 (10)	0.466^a^
Cardiac insufficiency	1 (7.70)	3 (13.30)	0 (0)	0.264^a^
Autoimmune disease	1 (7.70)	0 (0)	0 (0)	0.253^a^
**Physical examination**				
T Max (°C)	38.55±1.45	37.75±1.22	NA	0.125^b^
Respiratory frequency (times/min)	23.08±3.87	20(20–21)	NA	0.020^c^
Systolic pressure (mmHg)	125.17±31.56	120(115–128)	NA	0.272^c^
Diastolic blood pressure (mmHg)	74±14.09	72.60±10.03	NA	0.765^b^
Mean arterial pressure (mmHg)	84.05±29.78	89.93±8.18	NA	0.469^b^
Lung rales—no. (%)	10(76.92)	9(60.00)	NA	0.435^c^
Disorder of consciousness rales—no. (%)	3(23.08)	0 (0%)	NA	0.087^a^
**Laboratory results**				
WBC (×10^9^/L)	13.68±8.64	6.38±2.48	NA	0.016^b^
NE (×10^9^/L)	12.05±8.40	4.22±2.34	NA	0.002^b^
LY (×10^9^/L)	0.95±0.50	1.46±0.56	NA	0.022^b^
MO (×10^9^/L)	0.60±0.50	0.50±0.30	NA	0.547^b^
NE percentages (%)	84.72±8.12	60(57.80–64.40)	NA	<0.001^c^
LY percentages (%)	10.05±6.38	29.60(19.90–31.60)	NA	<0.001^c^
MO percentages (%)	4.53±2.50	7.88±3.07	NA	0.005^b^
NLR (%)	10.60 (4.30–22.90)	6.43±7.12	NA	0.001^c^
PLR (%)	348.08±320.11	127.30(83.60–448.27)	NA	0.188^c^
PLT (×10^9^/L)	228.38±110.76	218.13±66.91	NA	0.766^b^
CRP (mg/L)	144.78±139.20	32.60±31.39	NA	0.009^b^
PCT (μg/L)	0.54(0.32–7.48)	0.09±0.06	NA	0.002^c^
PaO_2_ (mmHg)	73.13±18.45	72.83±31.43	NA	0.978^b^
FiO_2_ (%)	44.36±17.82	21(21–25)	NA	0.003^c^
PaO_2_/FiO_2_	199.89±66.86	372.00±47.23	NA	<0.0001^b^
PaCO_2_ (mmHg)	39.82±10.32	36.06±4.51	NA	0.374^b^
SaO_2_ (%)	95.56±2.47	95.80±2.69	NA	0.875^b^
HCO_3_ (mmol/L)	25.81±6.20	24.26±4.67	NA	0.580^b^
Total cholesterol (mmol/L)	3.49±1.36	4.31±0.67	NA	0.020^b^
Triglyceride (mmol/L)	1.82(1.03–2.64)	0.84±0.20	NA	0.006^c^
**Detected pathogen**—no. (%)				
Bacteria	7(53.85)	2(13.33)	NA	0.042^a^
Virus	2(15.38)	8(53.33)	NA	0.055^a^
Fungus	4(30.77)	5(33.33)	NA	1.000^a^
**CURB-65**	1(1–0)	2(1–2)	NA	0.006^c^
**PSI**	93.85±28.70	67.40±30.51	NA	0.027^b^
**Hospitalization Days**	18.62±9.49	10.07±3.43	NA	0.003^b^
**30-day mortality-no. (%)**	4(30.77)	0(0)	0(0)	0.003^a^

**Note:** Descriptive statistics. Variables are expressed as numbers (percentages). Normally distributed continuous variables are expressed as means ± standard deviations (mean ± SD) and continuous nonparametric data are presented as medians and interquartile ranges (25th and 75th percentiles).

^a^ Chi-square test or Fisher’s exact test

^b^ Student’s t-test or analysis of variance with post-hoc Tukey HSD test

^c^ Mann-Whitney U or Kruskal-Wallis H test.

Abbreviations: BMI body mass index, COPD chronic obstructive pulmonary disease, WBC white blood cell, NE neutrophil, LY lymphocyte, MO monocyte, NLR neutrophil/lymphocyte ratio, PLR platelet-lymphocyte ratio, PLT blood platelet, CRP C-reactive protein, PCT procalcitonin, PaO_2_ partial pressure of oxygen, FiO_2_ Fraction of inspiration O_2_, SaO_2_ oxygen saturation, CURB-65 confusion, urea, respiratory rate, blood pressure, and age ≥65 years old, PSI pneumonia severity index, NA not applicable.

### Global lipidomic profiles of human serum

Serum lipidomic profiles of the 48 participants were generated using an untargeted lipidomic profiling analysis using HPLC-MS/MS. Overall, the lipids were categorized into six classes: glycerophospholipids (GP), glycerolipids (GL), sphingolipids (SP), prenol lipids (PR), sterol lipids (ST), and fatty acid (FA). A total of 509 lipid species were detected in the electrospray ionization positive (ESI+) mode. The top three dominant classes comprised over 99% of the total lipid signal, including GP (44.78%), GL (38.19%), and SP (16.06%). The lipid profile of each of the three groups was different. Compared with the NC group, the abundance of zymosterol ester, dihexosylceramide (Hex2Cer) in the CAP group was significantly increased (*p*<0.01, *p*<0.01, respectively,) while the abundances of lysophosphatidylcholine (LPC), diacylglycerol (DG), and PE showed a statistically significant decrease (all *p*<0.01). As the disease gradually worsened, the abundance of the eight lipid subclasses detected in SCAP patients decreased significantly, including LPC (*p*<0.01), LPE (*p*<0.01), hexosylceramide (HexCer) (*p*<0.01), Hex2Cer (*p*<0.05), trihexosylceramide (Hex3Cer) (*p*<0.05), ganglioside GM3 (GM3) (*p*<0.05), campesterol ester (CmE) (*p*<0.05), and cholesteryl ester (ChE) (*p*<0.0001) (S1 Table).

In the electrospray ionization negative (ESI-) mode, although 195 lipid species were detected, only the FAs were recognized more effectively. Only three classes of compounds were detected in the negative ion mode, namely, GP, SP, and FA. They accounted for 57.14%, 25.83%, and 17.03%, respectively. Subclasses such as PC (47.14%), sphingomyelin (SM, 22.35%), and free fatty acid (FFA, 17.03%) contributed 74.05% to the total lipid signal. Similarly, there are numerous differences in serum lipid profiles between NSCAP, SCAP, and NC. The abundance of Hex2Cer in the CAP group was significantly higher than in the NC group (*p*<0.001), while the abundance of LPC, PI, and PE in this group showed the opposite trend (*p*<0.001, *p*<0.05, *p*<0.05, respectively). At the same time, compared with NSCAP, the abundance of LPC, HexCer, Hex3Cer of SCAP group decreased (*p*<0.01, *p*<0.05, *p*<0.05, respectively), while the abundance of PE increased (*p*<0.05) (S1 Table).

### Multivariate models established by untargeted lipidomics analysis

**Fig 1:**
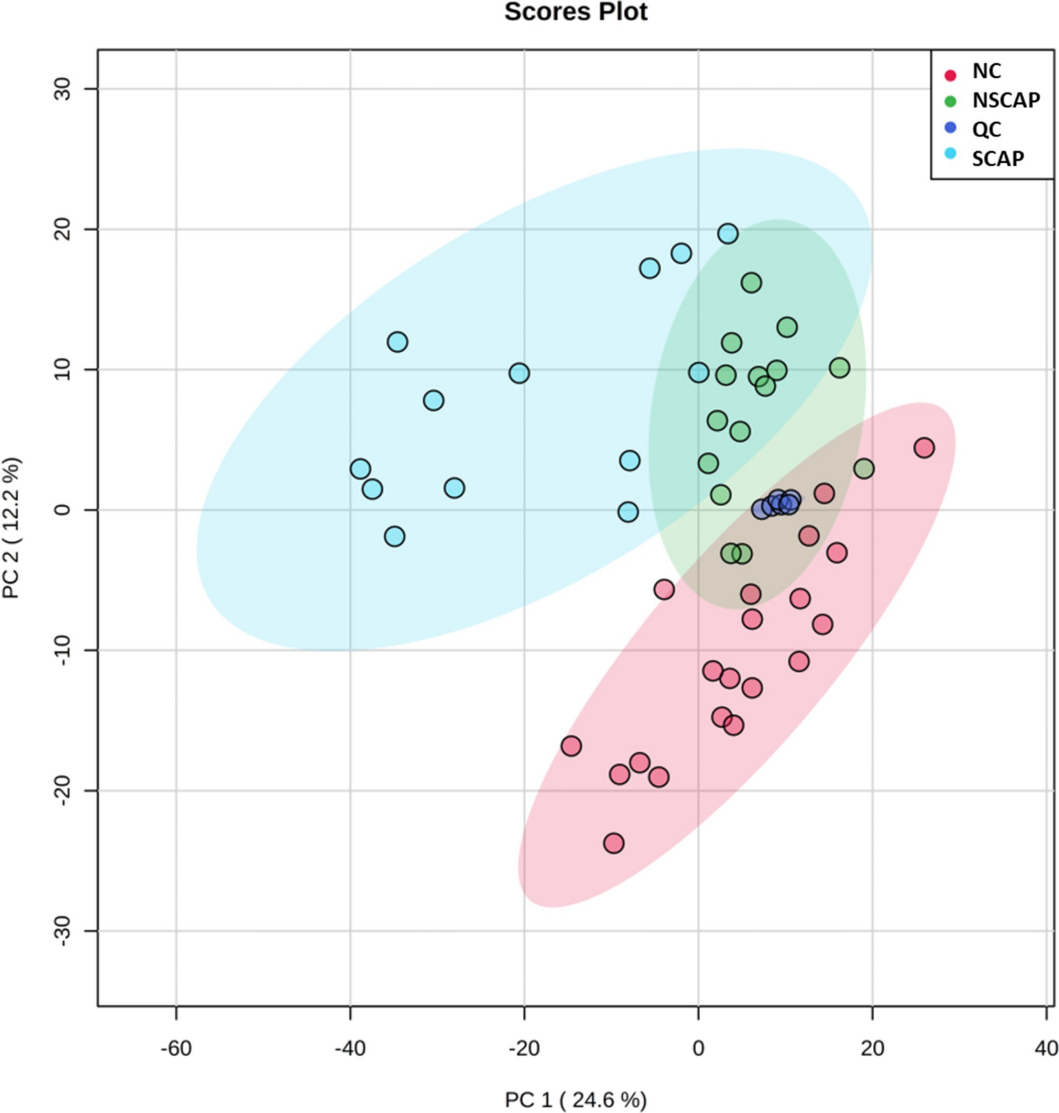
PCA scores plot of lipidomic profiles in CAP group (including NSCAP and SCAP) and NC. Blue, severe CAP (SCAP); Green, non-severe CAP (NSCAP); Red, non-CAP control (NC), Navy blue, quality control (QC) samples; Black, CAP. No sample was placed outside the ellipse that describes the 95% CI of Hotelling’s T-squared distribution. The three groups of lipid profiles can be clearly distinguished.

PCA is an effective approach for classifying data, detecting outliers, and validating the stability and reproducibility of an analytical method. All identified lipids were subjected to a PCA using MetaboAnalyst 4.0 to explore the major effects that potentially drive the differences in lipid profiles in CAP patients (NSCAP and SCAP) and NC (Fig 1). The optimal PCA model contained eight components. The principal component (PC) 1(the first component) explained the largest variation in the variable (24.6%), followed by PC2 (12.2%), etc. Hence, the scores scatter plot composed of PC1 and PC2 shows the positional relationship between variables. The value of R2 (cum) and Q2 (cum) (0.674 and 0.449), respectively, represented the fit and predicted power of the model. All QC samples were tightly clustered, indicating that this instrument has good repeatability and stability. As shown in Fig 1A, no obvious outlier was detected in all serum samples. Importantly, the obvious intra-group clustering and inter-group separation between the three groups suggested that whether CAP or SCAP, the patient’s serum lipid profile has changed significantly.

OPLS-DA analysis identified the biggest variation in lipid profiling using a few orthogonal latent variables. To further eliminate the interference factors of the disease and maximize the extraction of the information on the differences in lipid mass spectra between the different groups, we used a supervised clustering method to verify the OPLS-DA model of the serum samples of the patients. To prevent overfitting, we performed a permutation test (500 iterations) on those models. The OPLS-DA score plot showed obvious a discriminatory trend between both the CAP group versus the NC, NSCAP versus NC, SCAP versus NC, and NSCAP versus SCAP groups (S1 Fig in S4 File). The CV-ANOVA *p*-values for all the models were less than 0.0001, indicating that all the differences between the groups were significant. The Q2Y of all models was higher than 0.9, which revealed the model had an excellent interpretation ability and superior predictive power (Q2>0.5) (Table 2). After the 500-it iteration of the permutation test, the R2 and Q2 values were less than the original model (Table 2), and the Q2 regression line was less than 0 in the Y-axis intercept (S2 Fig in S4 File), which proved that the model was robust and there was no overfitting. Notably, in the OPLS-DA model, the predictability of separating the SCAP group from the NC group (Q2 = 0.848) was better than separating the NSCAP group from the NC group (Q2 = 0.703). That is, compared with the NSCAP group, more changes were observed in the SCAP group with a better isolation from the NC group.

**Table 2:** Evaluation parameters of our OPLS-DA model.

Group	Evaluation parameters of OPLS-DA original model					Permutation test	
	PC ^a^	R2X	R2Y	Q2(cum)	CV-ANOVA (*p*)	R2	Q2
CAP vs NC	1+4+0	0.476	0.979	0.685	0.000001	0.913	-0.566
NSCAP vs NC	1+2+0	0.329	0.947	0.703	0.00000257	0.845	-0.489
SCAP vs NC	1+2+0	0.448	0.964	0.848	0.00000000172	0.790	-0.509
NSCAP vs SCAP	1+2+0	0.366	0.964	0.536	0.00757053	0.847	-0.386

^a^: PC The number of principal components in this model, which represents the number of predicted components plus the number of orthogonal components.

Abbreviations: CAP, community-acquired pneumonia; NC, non-CAP control; NSCAP, non-severe CAP; SCAP, severe CAP.

### Differential abundance of lipids associated with CAP disease and acute exacerbations

To identify lipids with significant abundance changes between the CAP groups versus the NC and NSCAP versus SCAP groups, we normalized the relative peak intensity data of all lipids detected and performed T-test for the univariate analysis. A total of 295 lipids were statistically different in abundance (Benjamini-Hochberg adjusted *p*-value (FDR) < 0.05) between the CAP and NC. The VIP score was used to quantify the contribution of each lipid level to the overall separation between the two groups in the OPLS-DA model. There were 226 lipids with VIP values greater than 1 between the CAP and NC. Compared with NSCAP and SCAP simultaneously, 297 lipids had statistical differences in abundance (FDR<0.05), and VIP of 246 lipids exceeded 1.

**Fig 2:**
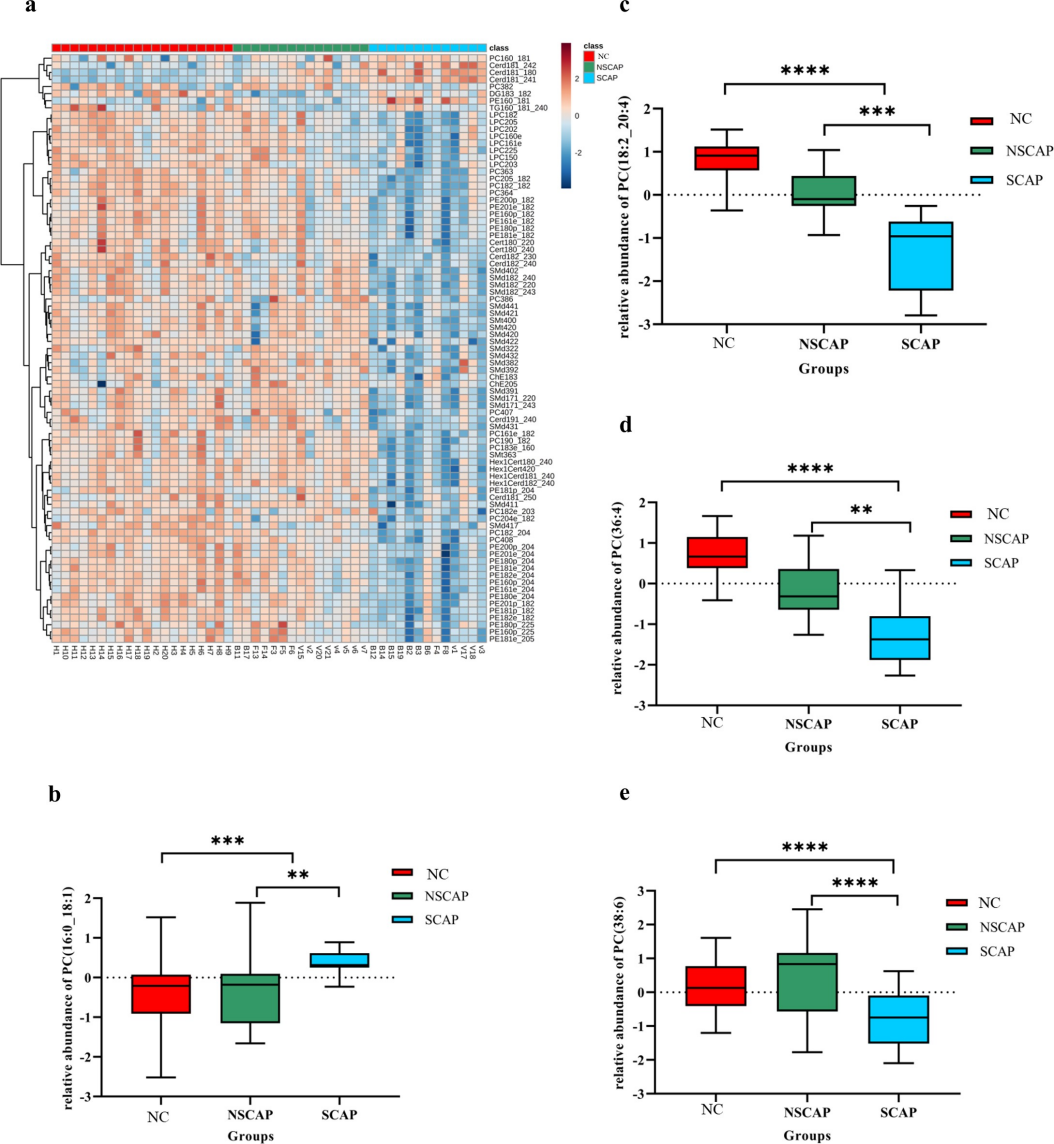
The relative abundance of different lipids changed in the three groups of samples. Red, green, and blue represent NC, NSCAP, and SCAP, respectively. **(a)** Hierarchical cluster heatmap of the relative abundance of 83 lipids in CAP (including NSCAP and SCAP) compared to NC. Distance measure: Pearson; Clustering algorithm: ward. Row represents lipids and column represents serum samples. Red, green, and blue represent NC, NSCAP, and SCAP, respectively. Light blue indicates lower relative abundance, while greater brown indicates higher intensity of lipids. **(b-e)** Changes in the relative abundance of PC (16:0_18:1), PC (18:2_20:4), PC (36:4) and PC (38:6) in serum between CAP group versus NC and NSCAP versus SCAP group. * *p*<0.05, ** *p*<0.01, *** *p*<0.001, **** *p*<0.0001.

Interestingly, we found that not only did the relative abundance of many lipids were different between the NC and CAP groups, but the levels of these lipids also changed significantly as the condition of CAP deteriorated (Fig 2A). To screen out the highest potential lipid that could distinguish CAP from NC and assess the severity at the early stage, we set the comparisons between the groups (CAP vs NC and NSCAP vs SCAP) to meet VIP> 1 and FDR <0.05 as the screening criteria. A total of 83 lipids met the screening criteria both in the comparison of the CAP versus HC and NSCAP versus SCAP groups. Remarkably, the relative levels of most of the differential lipids were continuously up-regulated or down-regulated in the CAP and SCAP group (S2 Table).

### Receiver Operating Characteristic (ROC) curve analysis

**Fig 3:**
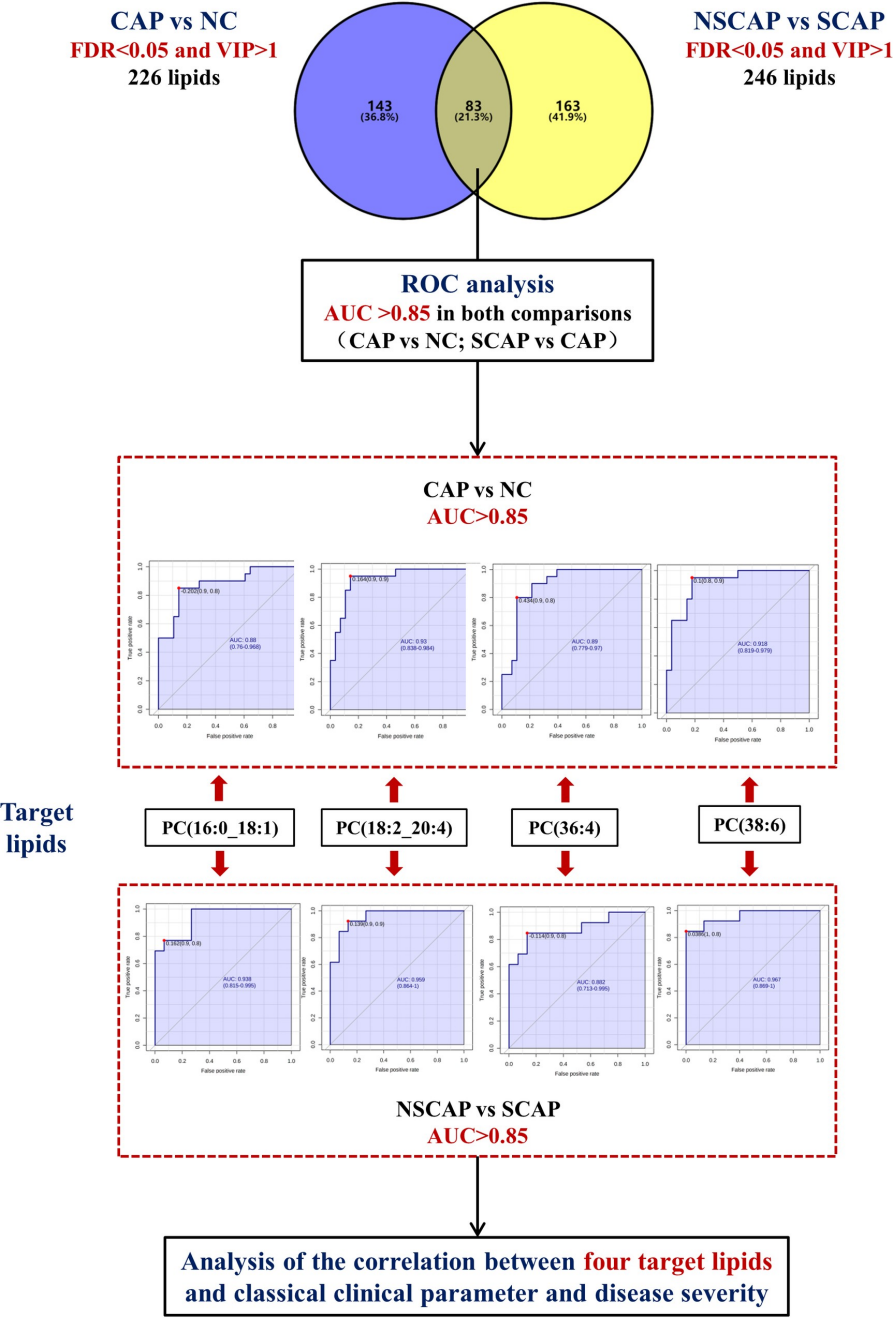
Flow chart of target differential lipid screening process. CAP, Community-acquired pneumonia; NC, Non-CAP controls; NSCAP, Non-severe CAP; SCAP, Severe CAP; VIP, variable importance on projection; ROC, Receiver operating characteristic; AUC, area under the curve.

ROC analysis was performed to investigate whether the abundance of screened lipids could be efficiently utilized for building a sensitive biosignature of severe status in CAP. We further selected lipids with an AUC greater than 0.85 in both comparisons (CAP vs NC; SCAP vs CAP) as target lipids (Fig 3). There were four lipids in total, PC (16:0_18:1), PC (18:2_20:4), PC (36:4), and PC (38:6) (Table 3), which were screened out. Furthermore, the relative increase in the AUC (95% CI) for all the four lipids were superior to PSI (0.749, 0.550–0.892) and CURB-65 (0.772, 0.575–0.908) for discriminating the SCAP group from the CAP group (S3 Table). Simultaneously, the relative abundance of PC (18:2_20: 4), PC (36:4), and PC (38:6) was significantly lower in the CAP group than in the NC group, and their abundance continued to decrease as the disease worsened. However, the relative abundance of PC (16:0_18:1) showed the opposite trend (Fig 2B–2E). The multiple logistic regression analysis revealed that a combination of these four lipid had an AUC value of 0.952 (0.848–0.993), with 78.57% sensitivity and 100% specificity when distinguishing CAP patients from NC, indicating that they can serve as a lipid panel of potential biomarkers for diagnosing CAP (S3 Table). However, when distinguishing between NSCAP and SCAP, the AUC (0.959 [0.808–0.998]) of the combined signature does not show better performance than a single lipid PC (38:6) (AUC = 0.959) (*p* > 0.05). Subsequently, the lipids with relatively lower AUC value (PC [16:0_18:1] and PC [36:4]) were combined as an indicator. It was found that the AUC of combined indicator (0.938 [0.779–0.994]) was superior to that of a single lipid (all *p* <0.0001), with sensitivity of 84.62% and specificity of 93.33% (S3 Table).

**Table 3:** Comparison results of four different lipids meeting the screening criteria.

Accepted Description	Ion Formula	CAP vs NC				SCAP vs NSCAP			
		Trend	VIP	*p*-value	AUC	Trend	VIP	*p*-value	AUC
PC(16:0_18:1)	C44 H85 O10 N1 P1	↑	1.269	<0.001	0.879	↑	1.179	<0.010	0.933
PC(18:2_20:4)	C48 H83 O10 N1 P1	↓	2.175	<0.00001	0.927	↓	1.747	<0.010	0.954
PC(36:4)	C44 H81 O8 N1 P1	↓	1.975	<0.00001	0.888	↓	1.389	<0.010	0.877
PC(38:6)	C46 H81 O8 N1 P1	↓	2.182	<0.00001	0.914	↓	1.863	<0.010	0.959

**Abbreviations:** CAP, community-acquired pneumonia; SCAP, severe CAP; NSCAP, non-severe CAP; NC, non-CAP control; PC, phosphatidylcholine; VIP, variable importance on projection value; ↑ Up-regulation ↓ Down-regulation.

### Correlation between the levels of four target lipids and clinical indicators

Since these clinical indicators showed abnormal distributions, Spearman’s rank correlation test was applied to further explore whether the relative abundance of the four target lipids were correlated with the clinical parameters: WBC, NE, LY, LY (%), MO (%), and NE (%) in serum, CRP, PCT, FiO_2_, PaO_2_/FiO_2_, CURB-65, and PSI. Owing to the lack of laboratory test data in the NC, we calculated the correlation between clinical indicators and the relative level of four lipids in the NSCAP and SCAP groups (S3 Fig in S4 File; S4 and S5 Tables).

**Fig 4:**
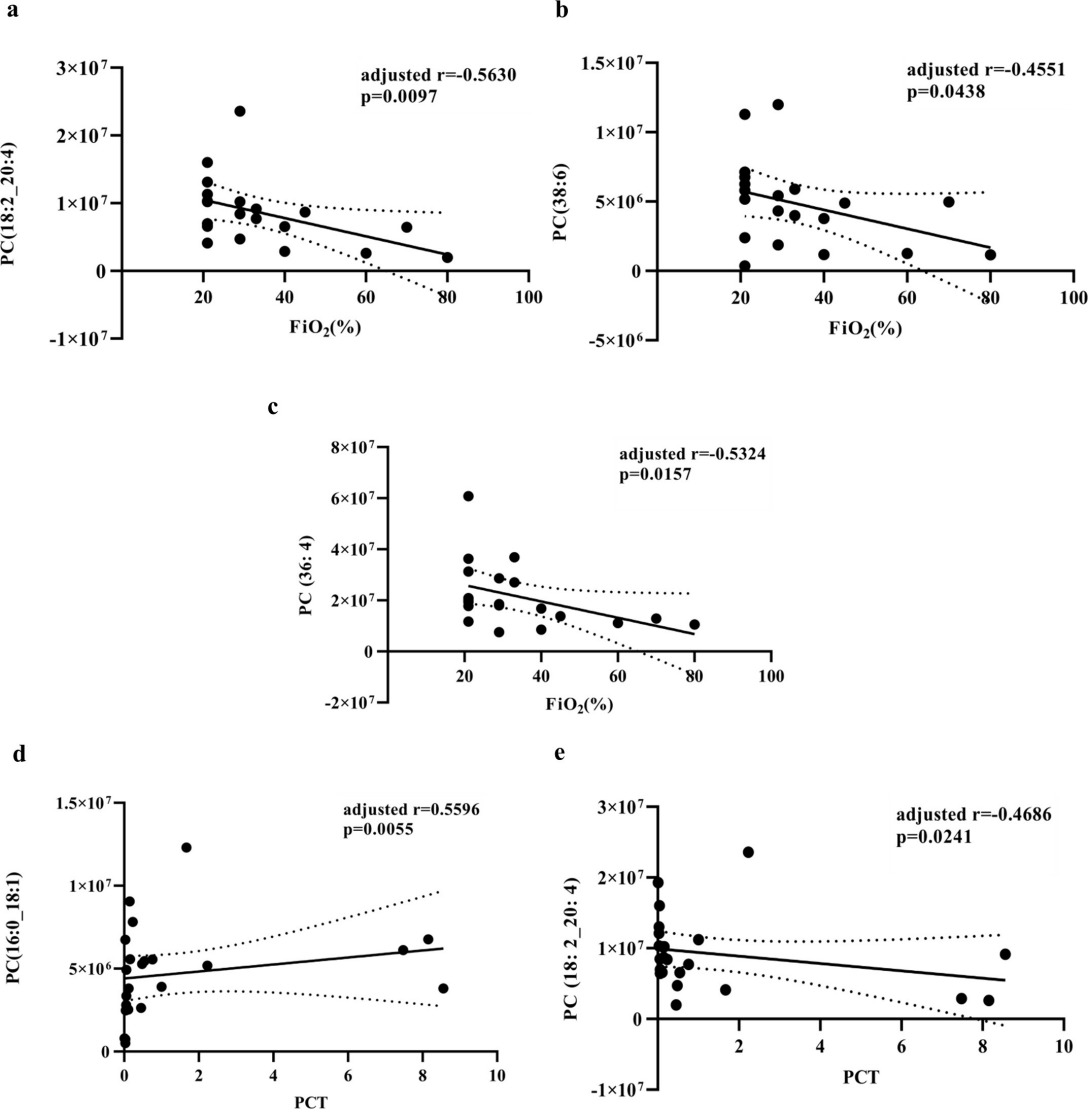
The relative levels of PC (16:0_18:1), PC (18:2_20:4), PC (38:6), and PC (36:4) show significantly correlations with clinical indicators. **(a-c)** The relative levels of PC (18:2_20:4) PC (38:6) and PC (36:4) were inversely correlated to FiO_2_ of patients with CAP. **(d)** The abundance of PC (16:0_18:1) had a positive linear relationship with PCT **(e)** The abundance of PC (18:2_20:4) was negatively correlated with PCT of CAP patients. Solid black line, the fitted regression line. Area within the dotted line lines, the 95% confidence intervals.

According to the results of the correlation analysis, the relative abundances of the four target lipids (PC [16:0_18:1], PC [18:2_20:4], PC [36:4], and PC [38:6]) were related to indices of infection. Considering the strong co-linearity between those lipids, an MLR analysis was conducted to evaluate the biochemical indices, which were independently correlated to lipid abundance. Finally, we identified that decreasing relative levels of PC (18:2_20:4), PC (38:6), and PC (36:4) were negatively related to FiO_2_ after *p* value adjustment. In addition, change in the relative abundance of PC (18:2_20:4) was inversely correlated with PCT, while the level of PC (16:0_18:1) had a positive linear relationship with PCT (Fig 4).

### The relative abundance of target lipids are associated with the prognosis of patients with SCAP

The AUCs for PC (16:0_18:1) and PC (18:2_20:4) were 0.885 and 0.954 for discriminating non-survivors from patients with CAP, respectively (*p* < 0.001 for both comparisons) (Table 4). The optimal normalized relative abundance threshold for predicting death was 0.392 of PC (16:0_18:1), with a sensitivity of 100% and specificity of 79.17%. While patients with PC (18:2_20:4) abundance <0.097 exhibited a noticeable increase in the risk of death, this threshold yielded sensitivity and specificity of 92.31% and 86.67%, respectively, for prediction of 30-day mortality (Table 4).

**Table 4:** Area under the curve (AUC) and thresholds for discriminating non-survivors from patients with CAP.

	Threshold	Sensitivity (%)	Specificity (%)	AUC	*p* value	95% CI	
						Lower limit	Higher limit
PC (16:0_18:1)	> 0.392	100	79.17	0.885	< 0.0001	0.708	0.974
PC (18:2_20:4)	≤ 0.353	100	45.83	0.708	0.1312	0.507	0.863
PC (36:4)	≤ -0.155	100	62.5	0.771	0.003	0.574	0.907
PC (38:6)	≤ 0.147	100	58.33	0.760	0.0099	0.563	0.9
Four lipids combined	--	100	79.17	0.875	<0.0001	0.695	0.969
PC (18:2_20:4)+ PC (36:4)+ PC (38:6)	--	100	66.67	0.792	0.0007	0.597	0.921
CURB-65	> 1	75	75	0.802	0.0057	0.609	0.927
PSI	> 114	50	91.67	0.625	0.5246	0.423	0.799

**Abbreviations:** CAP, community-acquired pneumonia; CURB-65, confusion, urea, respiratory rate, blood pressure, and age ≥65 years old score; PSI, Pneumonia Severity Index score.

**Fig 5:**
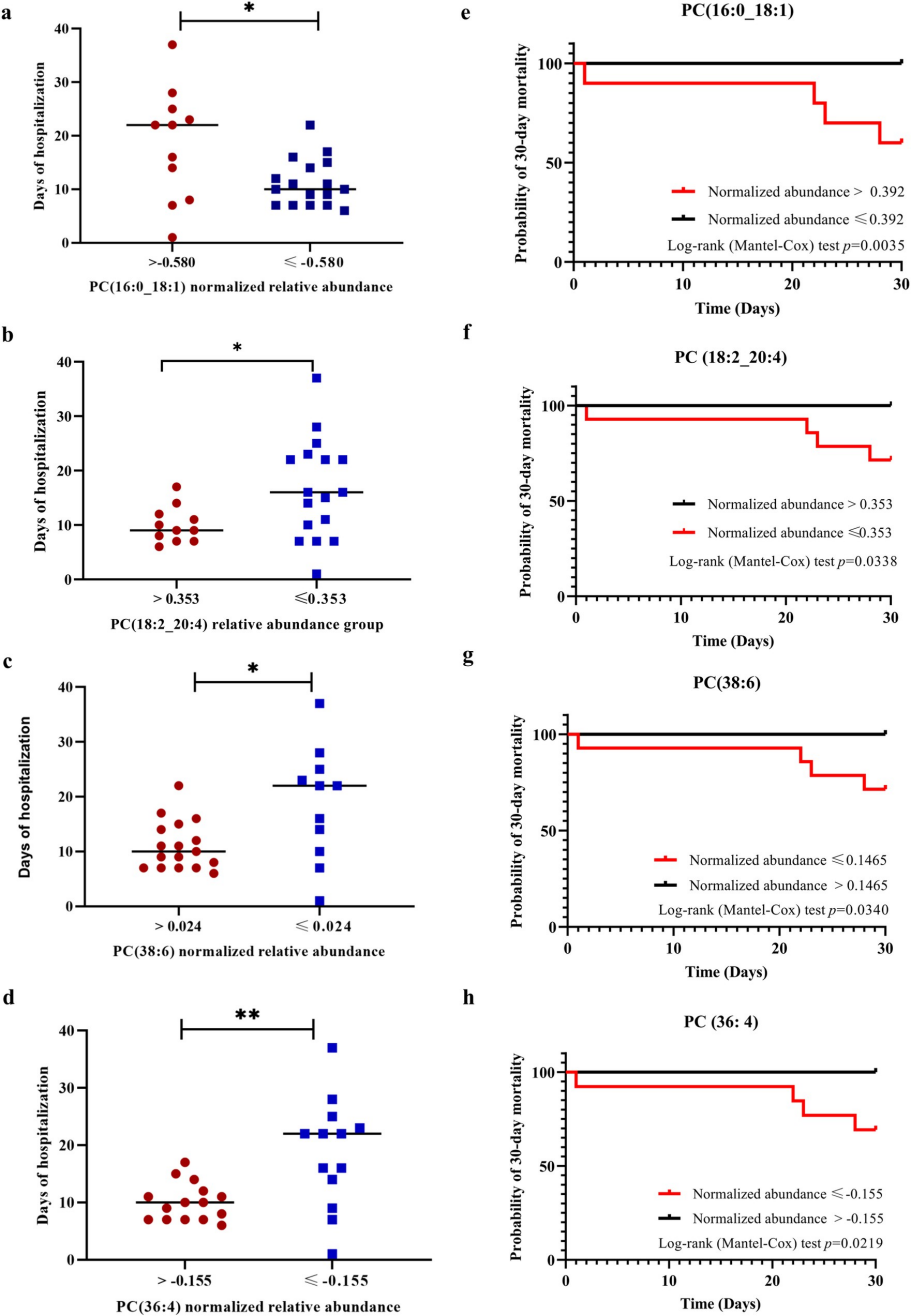
Comparison of the days of hospitalization between the higher abundance group and lower abundance group of PC (16:0_18:1), PC (18:2_20:4), PC (36:4), PC (38:6) and Kaplan–Meier analysis of 30-day mortality in patients with SCAP. (a-d) There were statistical differences in the days of hospitalization between the high abundance and low abundance groups of PC (16:0_18:1), PC (18:2_20:4), PC (36:4) and PC (38:6). Red dots, high abundance group; Blue squares, low abundance group. * *p*<0.05, ** *p*<0.01 (e-h) There were statistically significant differences in 30-day mortality between the high abundance group and the low abundance group of PC (16: 0_18: 1) (e), PC (18:2_20:4) (f), PC (36: 4) (g) and PC (38:6) (h).

The optimal threshold values of the ROC analysis were used as the cut-off to regroup to higher abundance group (normalized relative abundance was higher than cut-off value) and lower abundance group (normalized relative abundance was less than cut-off value) of CAP patients. Further, we compared the length of hospital stay of patients with higher and lower abundance groups. The results showed that patients with an elevated level of PC (16:0_18:1) (normalized relative abundance >-0.580) had significantly longer duration of hospital stays (*p*<0.05). Moreover, with the gradual decrease of serum PC (18:2_20:4), PC (36:4), and PC (38:6) levels, the length of hospitalization days of patients also increased significantly (all *p*<0.05, Fig 5A–5D). The Kaplan–Meier curve showed that there were statistically significant differences in mortality between the higher abundance and lower abundance groups of PC (18:2_20:4), PC (16:0_18:1), PC (36:4), and PC (38:6) (*p* = 0.0035, 0.0338, 0.0340 and 0.0219, respectively) (Fig 5E–5H). The dynamic changes of the relative abundances of specific lipids in serum were closely related to the disease progression and prognosis.

## Discussion

In this study, we used UHPLC-MS/MS technology to conduct exploratory studies and describe lipid mass spectrometry characteristics in 48 human serum samples, including the 28 CAP and 20 NC. We found that the lipid profiles of the NC, NSCAP, and SCAP groups were significantly different in both positive and negative ion modes. The untargeted lipidomic analysis showed that CAP patients could be clearly discriminated from NC patients, as well as the NSCAP group from the SCAP group, which further suggested that CAP and disease exacerbation caused significant fluctuations in lipidomic biochemical homeostasis. We further selected four lipids with AUCs greater than 0.85 in both comparisons of the CAP versus NC and NSCAP versus SCAP groups, as target lipids. The molecular levels of PC (18:2_20:4), PC (36:4) and PC (38:6) were significantly negatively correlated with FiO_2_ after *p* value adjustment. The abundance of PC (18:2_20:4) was inversely correlated with the PCT level, while the level of PC (16:0_18:1) had a positive linear relationship with PCT.

Lipids account for 90% of the surfactants in the lungs, and disorders of surfactants during pneumonia may cause changes in lipid metabolism [20]. Korneev et al. reported that large amounts of lipid metabolites were produced by bacteria, and lipids were important for the structure and function of bacteria [21]. At the same time, studies have shown that lipids are important inflammatory mediators during infection, and changes in lipid metabolites have been observed in cases such as sepsis [22], bacteremia [23], and viral infections [24, 25]. The differences in the lipid profile of the NC, NSCAP, and SCAP groups found in this study may be related to these reasons. Arshad et al. observed a striking decrease in the phospholipid concentrations in acute CAP, which largely normalized with clinical recovery. The greatest changes were seen in PC, followed by LPC, SM, and Cer [26]. Thus, the level of phospholipids can serve as highly accurate biomarkers for the diagnosis of CAP. This finding is consistent with our research. Moreover, decreased blood phospholipid concentrations have been documented in other invasive bacterial infections [27]. Studies by Arshad et al. also confirmed that at least part of the observed reduction in plasma concentration might be pre-programmed at the level of infected cells due to the phospholipase activity [26]. Additional studies are still needed to unravel the causes and consequences of reduced phospholipid abundance in CAP.

We further strictly limited the criteria used to select the differential abundance of lipids in this study, based on the combination of VIP > 1 and FDR < 0.05, which was consistent with the methods used in some previous metabolomics studies [28, 29]. In combination with the ROC analysis, four lipid small molecules were finally selected as target lipids and combined with clinical indicators for further correlation analyses. Studies have shown that changes in the abundance of PC (16:0_18:1), PC (18:2_20:4), PC (36:4), and PC (38:6) are closely related to pathogen infection, membrane injury, and liver injury [30–32]. Fluctuations in the abundance of these lipids lead to the change of glycerophospholipid metabolism. The disorder of the glycerophospholipid metabolic network is closely related to the occurrence and development of many diseases, such as coronary heart disease [33], atherosclerosis [34], brain injury [35], pain, and inflammation [36]. Studying the dynamic changes of the molecular composition of glycerophospholipids helps explain the molecular mechanism of disease pathogenesis and progression.

Hence, PCT is currently considered an infection-related biomarker and can be used as a specific indicator of bacterial infection [37]. Remarkably, the degree of PCT elevation is closely related to bacterial load and infection severity [38]. In addition, studies have shown that the prognostic utility was substantially improved when combined phospholipid and PCT criteria were applied to 28-day mortality outcome predictions in ICU patients with severe sepsis or septic shock [39]. The abundances of PC (16:0_18:1) and PC (18:2_20:4) had significant linear correlation with PCT level in our study. The results suggested that plasma phospholipids not only have strong CAP biomarker potential but fluctuations in its levels may also be closely related to bacterial infections. Evaluation of bacterial diseases, viruses, or other pathogen categories may help decide treatments to stop or gradually upgrade the use of antibiotics. Langley et al.’s [40] research showed that profiles of specific metabolites measured on days 1 and 7 differed markedly between survivors and non-survivors of severe septic shock, and patients who did not survive within 90 days showed a marked decrease in PC species. Consistently, in this study, we found that as the relative abundance of PC (18:2_20:4), PC (36:4), and PC (38:6) decreased, and the hospitalization days significantly extended. Particularly, as the levels of PC (16:0_18:1), PC (36:4), and PC (38:6) decreased, 30-day mortality rates increased significantly.

This study had several limitations. Even though our results agree well with published evidence, this study is limited by the lack of an external validation cohort and the relatively small sample sizes. Further research is, therefore, needed before the identified biomarkers can be advanced to clinical application. In addition, only the serum lipid relative abundance was detected at the time of admission; dynamic and follow-up changes (in response to treatment) were not investigated. The effects of changes in lipid expression during the pathogenesis of CAP should be further investigated. Moreover, multi-omics data are urgently needed to be integrated into the CAP study to further support the development of precision medicine.

## Conclusions

In conclusion, we demonstrated that lipidomic approaches based on HPLC-MS/MS could be used successfully to reveal changes in lipid abundance in CAP and establish a metabolite signature related to disease severity. It helps to adjust the treatment plan for the specific disordered lipid characteristics and related disease states exhibited by the patients.

## Supporting information

S1 File Questionnaire for the 30-day outcome of patients with community-acquired pneumonia (original language and English). (DOCX)

S2 File Supplemental methods for Ultra High-Performance Liquid Chromatography-Mass Spectrometry (UHPLC-MS/MS) analysis. (DOCX)

S3 File MS/MS spectra for assigning the lipid molecules. (DOCX)

S4 File S1 Fig. Orthogonal partial least squares discriminant analysis (OPLS-DA) of serum samples in the CAP group (including NSCAP and SCAP) and NC. (a) OPLS-DA score plot discriminates all CAP patients versus NC (b) OPLS-DA score plots of NSCAP versus NC group. (c) OPLS-DA score plots of SCAP versus NC. (e) OPLS-DA score plots of NSCAP versus SCAP. The model of OPLS-DA reflect good separation trends among SCAP, NSCAP and NC. Blue, severe CAP (SCAP); Green, non-severe CAP (NSCAP); Red, non-CAP control (NC), Black, CAP. S2 Fig. Permutation tests of all the OPLS-DA models. All OPLS-DA models have been verified using permutation tests. Permutation verification was established after 500 iterations. The Permutations Plot helps to assess the risk that the current OPLS-DA model is spurious. The plot shows, for a selected Y-variable, on the vertical axis the values of R2 and Q2 for the original model (far to the right) and of the Y-permuted models further to the left. The horizontal axis shows the correlation between the permuted Y-vectors and the original Y-vector for the selected Y. The plot above strongly indicates that the original model is valid. The criteria for validity are: all blue Q2-values to the left are lower than the original points to the right or the blue regression line of the Q2-points intersects the vertical axis (on the left) at, or below zero. The permutation test of the OPLS-DA model constructed by CAP versus HC groups (a), NSCAP versus HC (b), SCAP versus HC (c) and SCAP versus SCAP (d). S3 Fig. Correlation between the target lipids and the clinical indicators. The color transition from dark blue to red indicates the correlation from low to high.
(DOCX)

S5 File Structural confirmations of the four target lipids. S1 Fig. Some representative MS/MS spectra to assign the lipid molecules. S2 Fig. Representative chemical structures and fragmentation of PC in positive mode and negative mode. Red lines displays collision induced fragments generated in negative mode and blue lines are fragments in positive mode. S3 Fig. Determination of the chemical structure of PC (16:0_18:1) in the serum extract using tandem mass spectrometry. S4 Fig. Determination of chemical structures of PC (18:2_20:4) in serum extract using tandem mass spectrometry. S5 Fig. Determination of chemical structures of PC (36:4) in serum extract using tandem mass spectrometry. S6 Fig. Determination of chemical structures of PC (38:6) in serum extract using tandem mass spectrometry.
(DOCX)

S1 Table Global serum lipidomic profiles of 48 patients generated through untargeted lipidomic profiling analysis using HPLC-MS/MS. (XLSX)

S2 Table The 83 lipids that discriminate the NC, NSCAP and SCAP groups. (XLSX)

S3 Table Areas under the curve (AUCs) and thresholds for all ROC analysis. (DOCX)

S4 Table Correlation analyses between the abundance of four target lipids for CAP and the clinical parameters—Spearman’s rank correlation coefficient. (XLSX)

S5 Table Correlation analysis between the abundance of four target lipids for CAP and the clinical parameters—*p* value. (XLSX)
