# Supplementary material for: Lipid profiles and differential lipids in serum related to severity of community-acquired pneumonia: A pilot study
Source: PLoS One. 2021 Mar 11;16(3):e0245770. doi: 10.1371/journal.pone.0245770 (PMC7951898; doi:10.1371/journal.pone.0245770)
Supplement: S1 File — (DOCX) [file pone.0245770.s001.docx]

**Questionnaire for the 30-day outcome of patients with community-acquired pneumonia**

**Basic patient information (doctors fill in according to medical records)**

Name: Gender: Age:

Medical record number: Department: Tel:

Admission time: Discharge time: Discharge diagnosis:

Telephone interview date:

**Telephone interview**

1. Patient outcome:

□ Condition improved (interview finished)

□ Re-admission after discharge (Go to question 2)

□ The patient has died (Go to question 5)

2. Re-admission time and treatment department: (Go to question 3)

3. Reason for re-admission: (Go to question 4)

4. Re-admission outcome:

□ Condition improved (interview finished)

□ Worsening (interview finished)

□ The patient has died (Go to question 5)

5. Time of patient death：

**社区获得性肺炎患者30天结局情况电话回访问券**

**患者基本信息（医生按病历填写）**

姓名： 性别： 年龄：

病历号： 科室： 联系电话：

入院时间： 出院诊断： 出院时间：

**电话回访问询内容：**

1. 患者结局：

□ 好转（问卷截止）

□出院后再次入院（转至问题2）

□死亡（转至问题5）

2. 再次入院时间及就诊科室：（转至问题3）

3. 再次入院原因：（转至问题4）

4. 再次入院结局：

□ 好转

□ 病情加重

□ 死亡（转至问题5）

5. 患者死亡时间：
