# Supplementary material for: Lipid profiles and differential lipids in serum related to severity of community-acquired pneumonia: A pilot study
Source: PLoS One. 2021 Mar 11;16(3):e0245770. doi: 10.1371/journal.pone.0245770 (PMC7951898; doi:10.1371/journal.pone.0245770)
Supplement: S2 File — (DOCX) [file pone.0245770.s002.docx]

**Untra high-performance liquid chromatography-mass spectrometry (UHPLC-MS/MS) analysis**

After the dried samples were reconstituted, taken 40µl of the suspension from each sample and added it to a 2mL chromatographic injection bottle for on-machine testing.

All serum samples were analysed using an Ultimate 3000 UHPLC (Dionex) system coupled to a Thermo Q-Exactive (Orbitrap) mass spectrometer (Thermo Fisher Scientific, San Jose, CA, USA). The detection was performed in the positive and negative ion scanning modes, respectively.

The detailed mass spectrometer parameters are as follows: spray voltage, 3.2 KV for positive and 2.8 KV for negative mode; source temperature, 320°C; sheath gas flow rate (arb), 35; aux gas flow rate (arb), 10; mass range (m/z), 240–2000 for positive and 200–2000 for negative; full MS resolution, 70, 000; MS/MS resolution, 17,500; top N, 10; stepped NCE, 15/25/35; duty cycle (s), ~1.2. Since full width at half maximum (FWHM) of chromatographic peaks in this approach is ~10 s, this gives each peak ~15 points in acquisition and provides reliable quantitation in this label free method.

LC condition: column: Cortecs, 2.1 mm ×100 mm; column chamber T(℃): 40℃; flow rate :0.25 mL/min; mobile phase A: 10Mm NH4 Ac 60%ACN 40% H_2_O; mobile phase A: 90% IPA 10% ACN; gradient: 2.5min-33%; 5min-45%; 6min-52%; 9min-58%; 12min-66%; 15min-70%; 19.5min-98%; 21min-98%; 29.5min-98%; 30min-33%; 35min-33%.
