# Supplementary material for: Lipid profiles and differential lipids in serum related to severity of community-acquired pneumonia: A pilot study
Source: PLoS One. 2021 Mar 11;16(3):e0245770. doi: 10.1371/journal.pone.0245770 (PMC7951898; doi:10.1371/journal.pone.0245770)
Supplement: S3 File — (DOCX) [file pone.0245770.s003.docx]

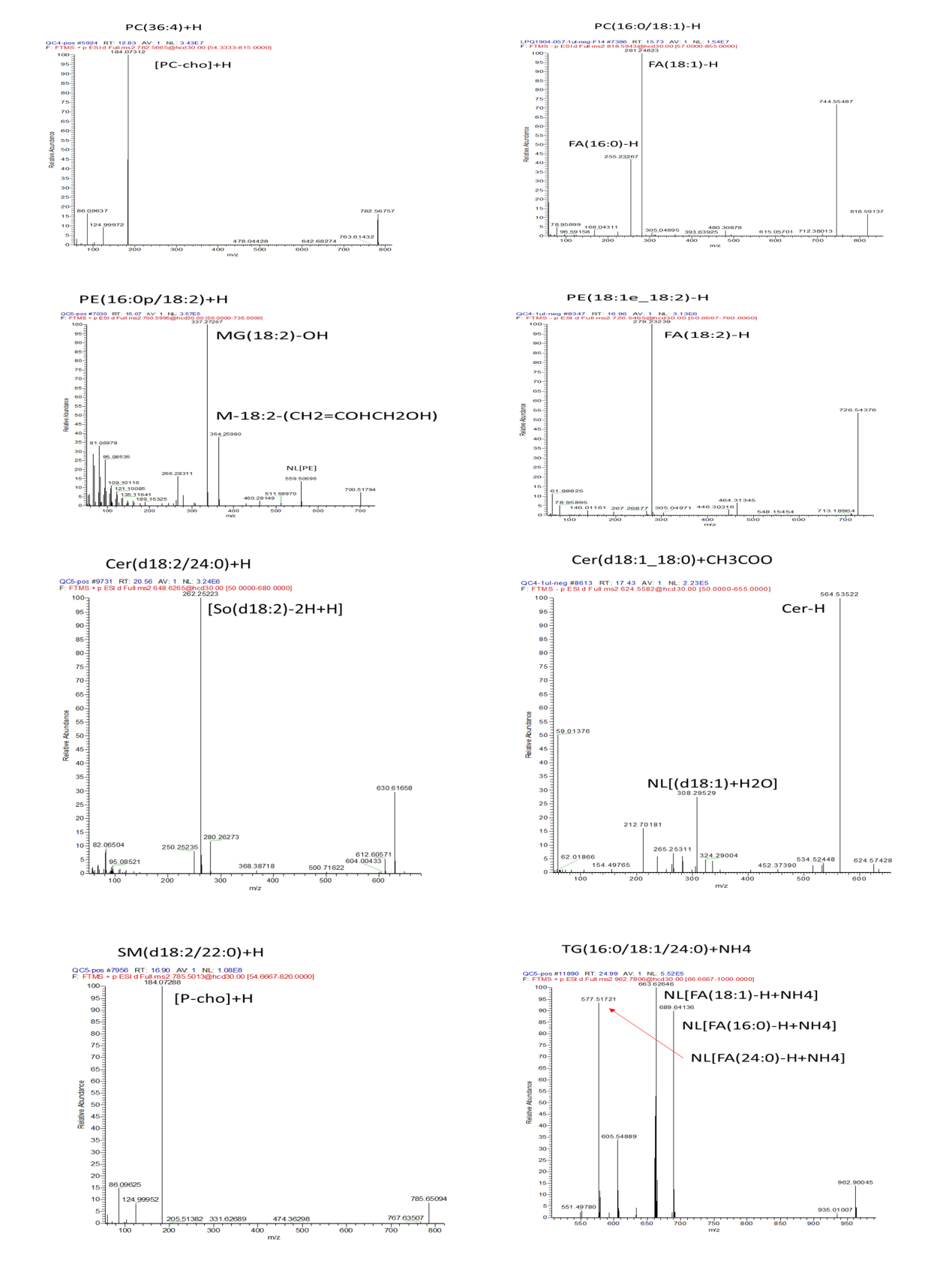


**S1 Fig.** Some representative MS/MS spectra to assign the lipid molecules.

**
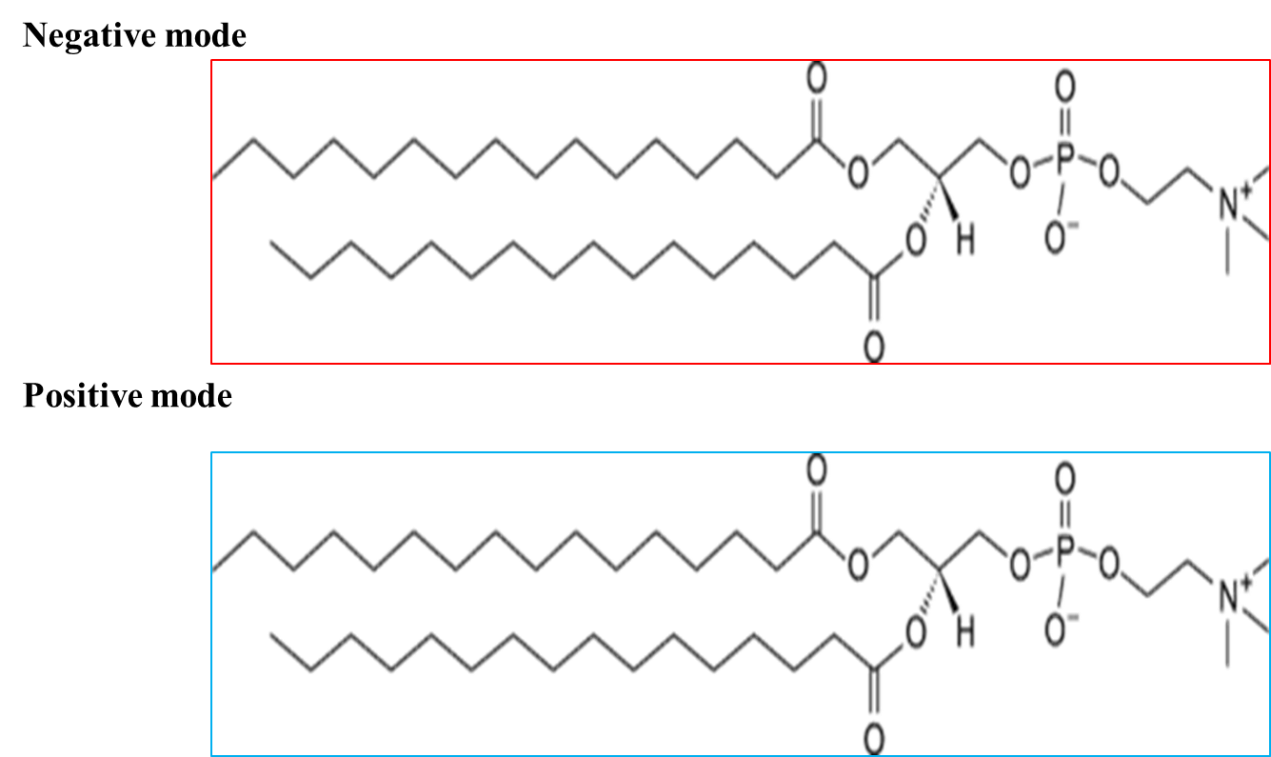
**

**S2 Fig. Representative chemical structures and fragmentation of PC in positive mode and negative mode.** Red lines displays collision induced fragments generated in negative mode and blue lines are fragments in positive mode**.**
