# Supplementary material for: Lipid profiles and differential lipids in serum related to severity of community-acquired pneumonia: A pilot study
Source: PLoS One. 2021 Mar 11;16(3):e0245770. doi: 10.1371/journal.pone.0245770 (PMC7951898; doi:10.1371/journal.pone.0245770)
Supplement: S4 File — S1 Fig. Orthogonal partial least squares discriminant analysis (OPLS-DA) of serum samples in the CAP group (including NSCAP and SCAP) and NC. (a) OPLS-DA score plot discriminates all CAP patients versus NC (b) OPLS-DA score plots of NSCAP versus NC group. (c) OPLS-DA score plots of SCAP versus NC. (e) OPLS-DA score plots of NSCAP versus SCAP. The model of OPLS-DA reflect good separation trends among SCAP, NSCAP and NC. Blue, severe CAP (SCAP); Green, non-severe CAP (NSCAP); Red, non-CAP control (NC), Black, CAP. S2 Fig. Permutation tests of all the OPLS-DA models. All OPLS-DA models have been verified using permutation tests. Permutation verification was established after 500 iterations. The Permutations Plot helps to assess the risk that the current OPLS-DA model is spurious. The plot shows, for a selected Y-variable, on the vertical axis the values of R2 and Q2 for the original model (far to the right) and of the Y-permuted models further to the left. The horizontal axis shows the correlation between the permuted Y-vectors and the original Y-vector for the selected Y. The plot above strongly indicates that the original model is valid. The criteria for validity are: all blue Q2-values to the left are lower than the original points to the right or the blue regression line of the Q2-points intersects the vertical axis (on the left) at, or below zero. The permutation test of the OPLS-DA model constructed by CAP versus HC groups (a), NSCAP versus HC (b), SCAP versus HC (c) and SCAP versus SCAP (d). S3 Fig. Correlation between the target lipids and the clinical indicators. The color transition from dark blue to red indicates the correlation from low to high. (DOCX) [file pone.0245770.s004.docx]

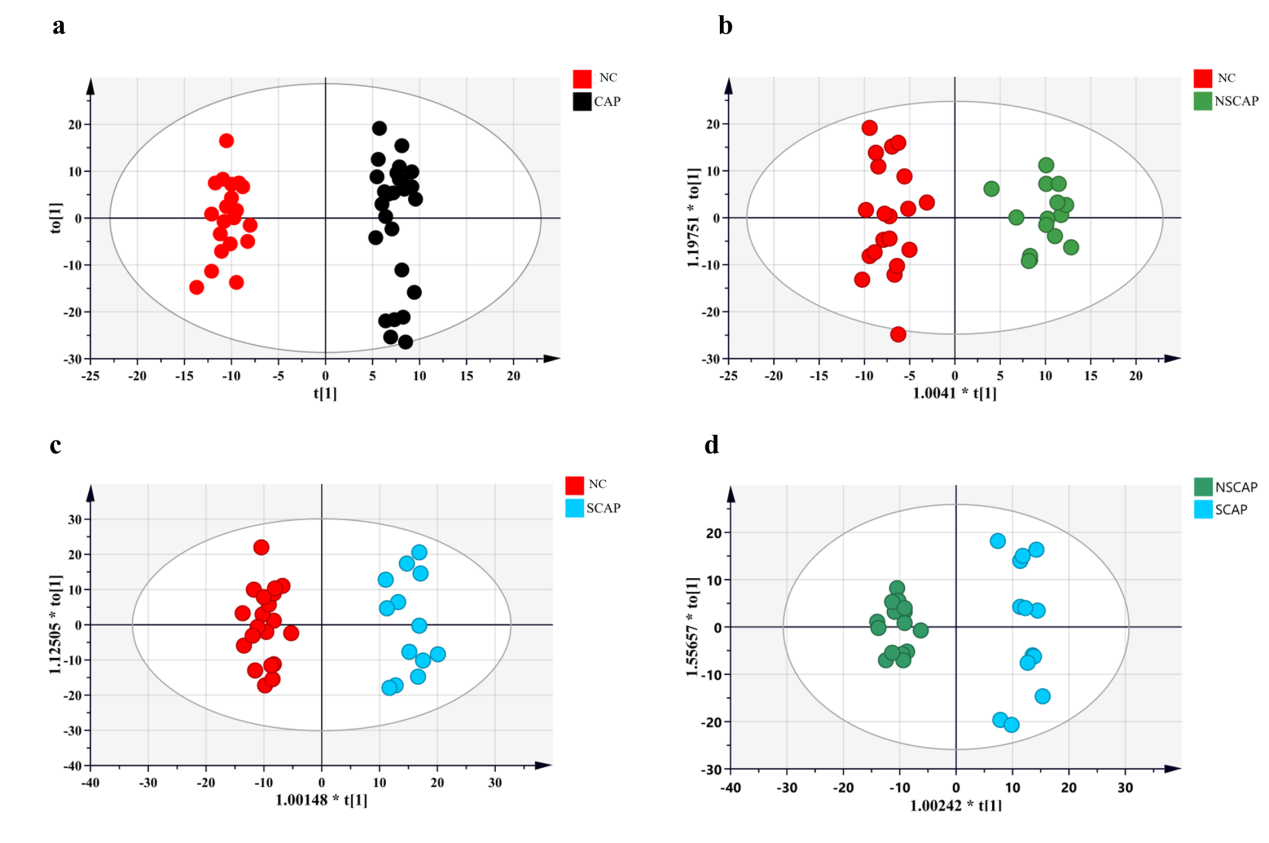


**S1 Fig.** **Orthogonal partial least squares discriminant analysis (OPLS-DA) of serum samples in CAP group (including NSCAP and SCAP) and NC.** (**a)** OPLS-DA score plot discriminates all CAP subjects versus NC (**b)** OPLS-DA score plots of NSCAP versus NC group. (**c**) OPLS-DA score plots of SCAP versus NC. (**e)** OPLS-DA score plots of NSCAP versus SCAP. The model of OPLS-DA reflect good separation trends among SCAP, NSCAP and NC. Blue, severe CAP (SCAP); Green, non-severe CAP (NSCAP); Red, non-CAP control (NC), Black, CAP.


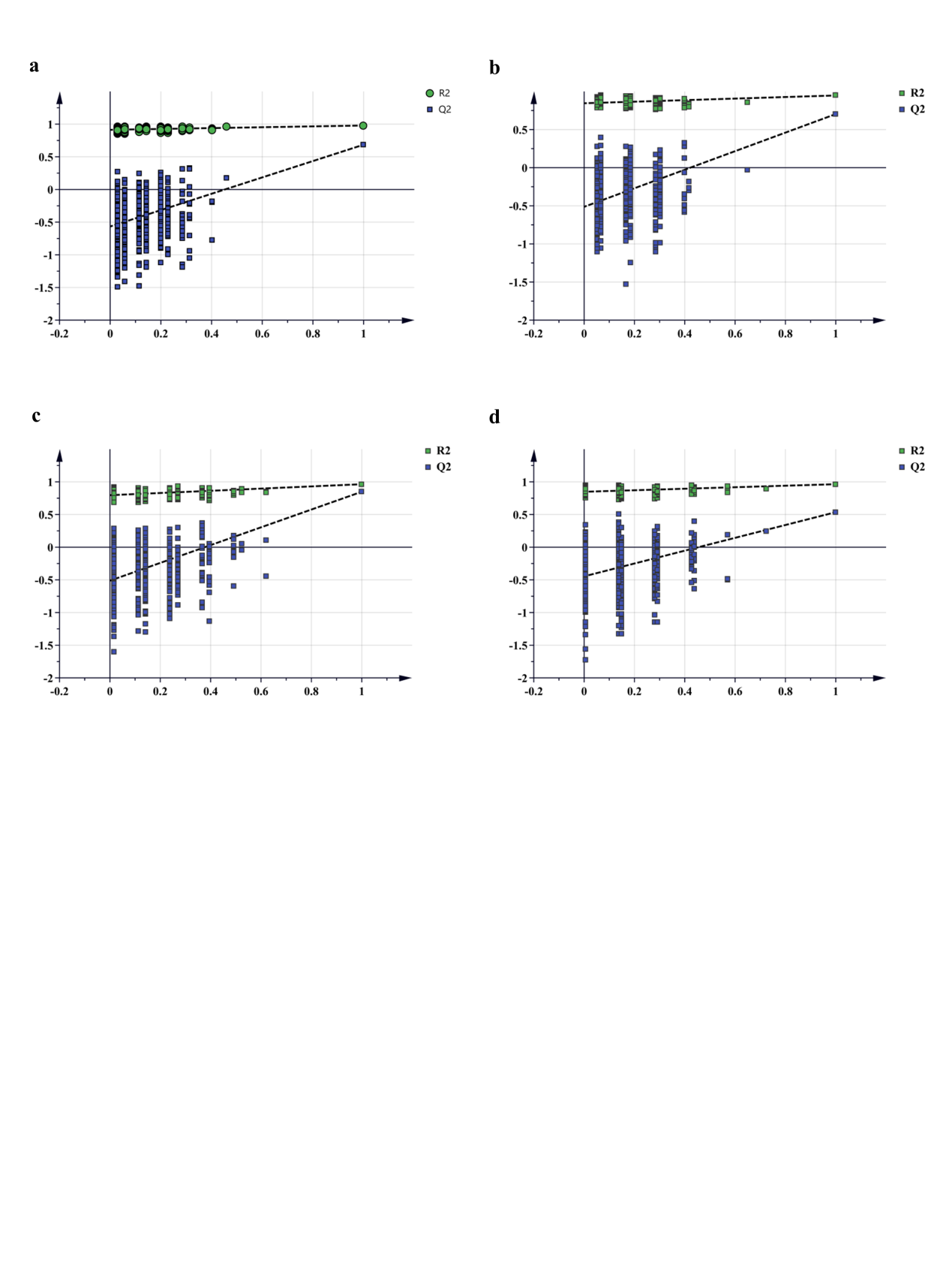


**S2 Fig. Permutation test of all OPLS-DA models.** Permutation verification established after 500 iterations. The Permutations Plot helps to assess the risk that the current OPLS-DA model is spurious. The plot shows, for a selected Y-variable, on the vertical axis the values of R2 and Q2 for the original model (far to the right) and of the Y-permuted models further to the left. The horizontal axis shows the correlation between the permuted Y-vectors and the original Y-vector for the selected Y. The plot above strongly indicates that the original model is valid. The criteria for validity are: all blue Q2-values to the left are lower than the original points to the right or the blue regression line of the Q2-points intersects the vertical axis (on the left) at, or below zero. The permutation test of the OPLS-DA model constructed by CAP versus HC groups **(a)**, NSCAP versus HC **(b)**, SCAP versus HC **(c)** and SCAP versus SCAP **(d)**.


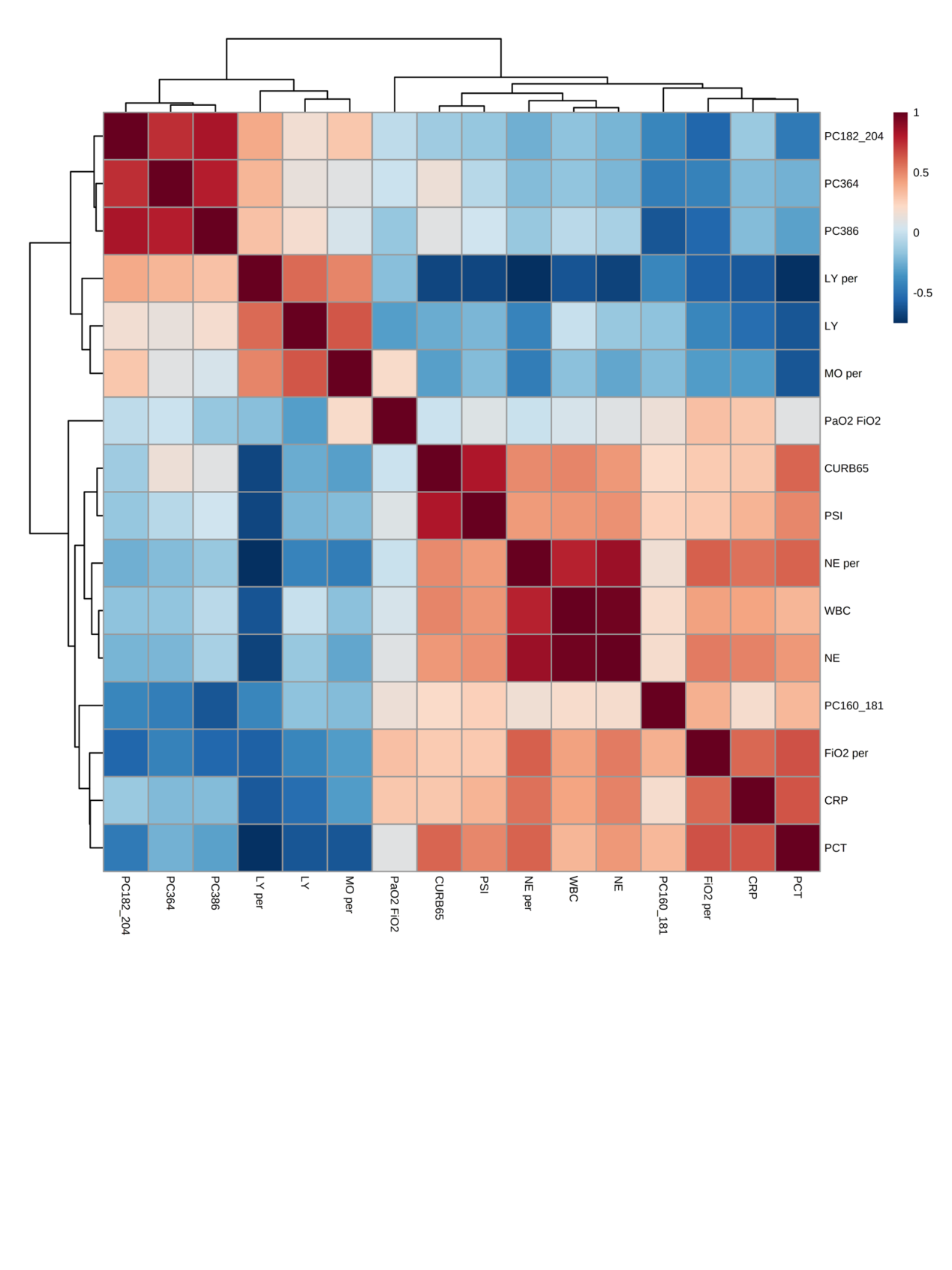


**S3 Fig. Correlation between putative biomarkers and clinical indicators.** The color transition from dark blue to red indicates the correlation from low to high.
