# Supplementary material for: Lipid profiles and differential lipids in serum related to severity of community-acquired pneumonia: A pilot study
Source: PLoS One. 2021 Mar 11;16(3):e0245770. doi: 10.1371/journal.pone.0245770 (PMC7951898; doi:10.1371/journal.pone.0245770)
Supplement: S5 File — S1 Fig. Some representative MS/MS spectra to assign the lipid molecules. S2 Fig. Representative chemical structures and fragmentation of PC in positive mode and negative mode. Red lines displays collision induced fragments generated in negative mode and blue lines are fragments in positive mode. S3 Fig. Determination of the chemical structure of PC (16:0_18:1) in the serum extract using tandem mass spectrometry. S4 Fig. Determination of chemical structures of PC (18:2_20:4) in serum extract using tandem mass spectrometry. S5 Fig. Determination of chemical structures of PC (36:4) in serum extract using tandem mass spectrometry. S6 Fig. Determination of chemical structures of PC (38:6) in serum extract using tandem mass spectrometry. (DOCX) [file pone.0245770.s005.docx]

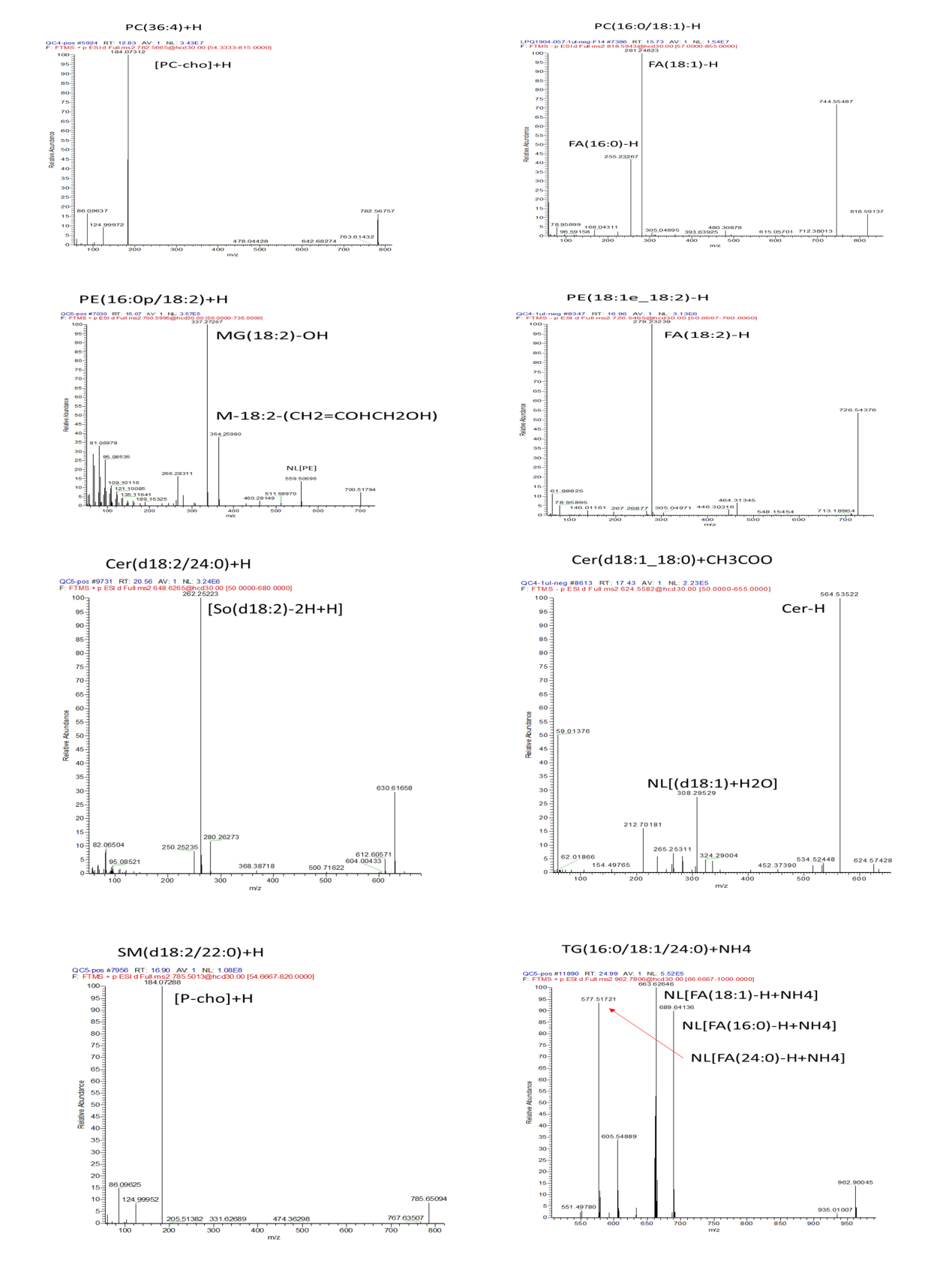


**S1 Fig.** Some representative MS/MS spectra to assign the lipid molecules.

**
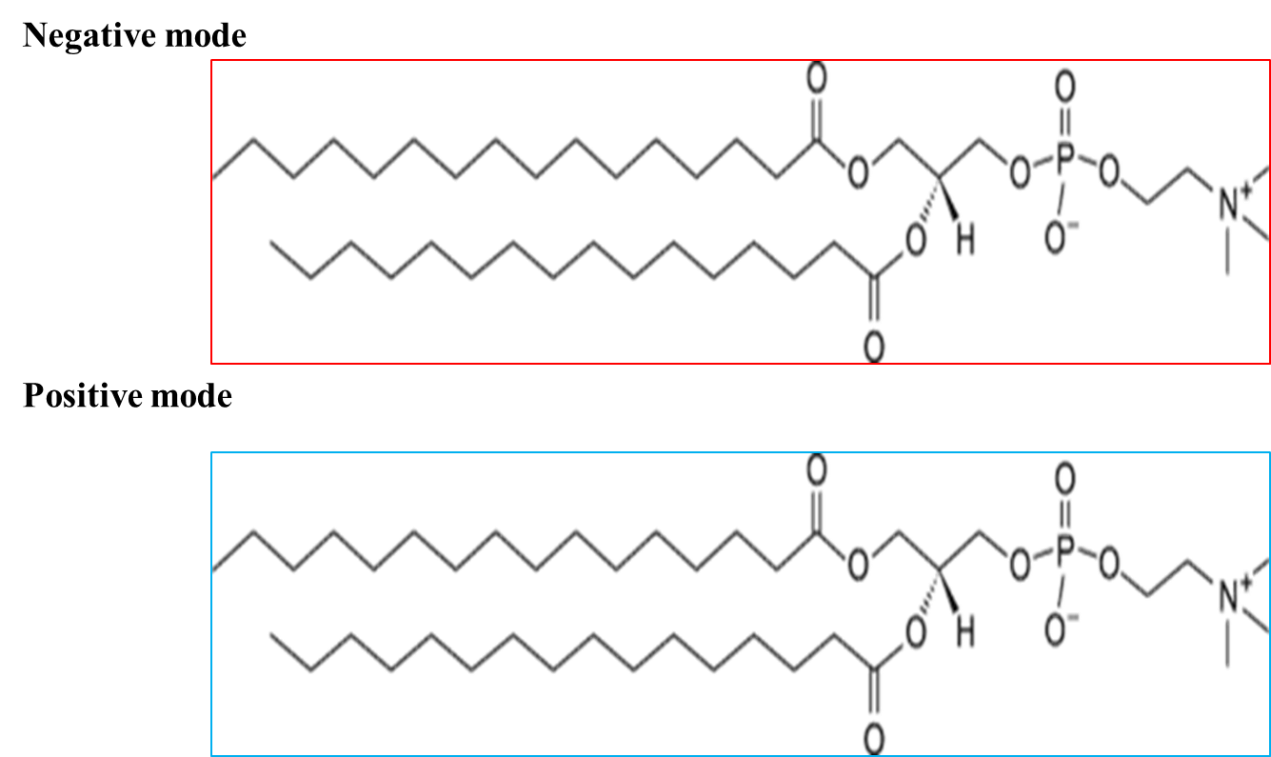
**

**S2 Fig. Representative chemical structures and fragmentation of PC in positive mode and negative mode.** Red lines displays collision induced fragments generated in negative mode and blue lines are fragments in positive mode**.**

FA(18:1)

FA(16:0)

**S3 Fig. Determination of chemical structures of PC(16:0_18:1) in serum extract by tandem mass spectrometry.**

FA(18:1)

FA(20:4)

**S4 Fig. Determination of chemical structures of PC (18:2_20:4) in serum extract by tandem mass spectrometry.**

PC-cho+H

**S5 Fig. Determination of chemical structures of PC (36:4) in serum extract by tandem mass spectrometry.**

**S6 Fig. Determination of chemical structures of PC (38:6) in serum extract by tandem mass spectrometry.**
