# Supplementary material for: Lipid profiles and differential lipids in serum related to severity of community-acquired pneumonia: A pilot study
Source: PLoS One. 2021 Mar 11;16(3):e0245770. doi: 10.1371/journal.pone.0245770 (PMC7951898; doi:10.1371/journal.pone.0245770)
Supplement: S3 Table — (DOCX) [file pone.0245770.s008.docx]

**S3 Table Areas under the curve (AUCs) and thresholds for all ROC analysis**

|  |  | Threshold | Sensitivity (%) | Specificity (%) | AUC | *p* value | 95% CI | |
| --- | --- | --- | --- | --- | --- | --- | --- | --- |
|  |  |  |  |  |  |  | Lower limit | Higher limit |
| CAP vs NC | PC(16:0_18:1) | >5.672 | 85.71 | 85.00 | 0.879 | <0.0001 | 0.752 | 0.955 |
|  | PC(18:2_20:4) | ≤0.390 | 85.71 | 95.00 | 0.927 | <0.0001 | 0.814 | 0.982 |
|  | PC(36:4) | ≤1.884 | 89.29 | 80.00 | 0.888 | <0.0001 | 0.763 | 0.960 |
|  | PC(38:6) | ≤-0.356 | 82.14 | 95.00 | 0.914 | <0.0001 | 0.797 | 0.976 |
|  | Four lipids combined | -- | 78.57 | 100.00 | 0.952 | <0.0001 | 0.848 | 0.993 |
| NSCAP  vs  SCAP | PC(16:0_18:1) | > -0.580 | 100 | 73.33 | 0.933 | <0.0001 | 0.772 | 0.993 |
|  | PC(18:2_20:4) | ≤ 0.097 | 92.31 | 86.67 | 0.954 | <0.0001 | 0.801 | 0.998 |
|  | PC(36:4) | ≤ -0.155 | 84.62 | 86.67 | 0.877 | <0.0001 | 0.698 | 0.97 |
|  | PC(38:6) | ≤ 0.024 | 84.62 | 100 | 0.959 | <0.0001 | 0.808 | 0.998 |
|  | Four lipids combined | -- | 84.62 | 100 | 0.959 | <0.0001 | 0.808 | 0.998 |
|  | PC(16:0_18:1)+ PC(36:4) | -- | 84.62 | 93.33 | 0.938 | <0.0001 | 0.779 | 0.994 |
|  | CURB-65 | > 1 | 61.54 | 93.33 | 0.772 | 0.0032 | 0.575 | 0.908 |
|  | PSI | > 71 | 76.92 | 73.33 | 0.749 | 0.0113 | 0.55 | 0.892 |
| Survivors  vs  Non-surv-ivors | PC(16:0_18:1) | > 0.392 | 100 | 79.17 | 0.885 | < 0.0001 | 0.708 | 0.974 |
|  | PC(18:2_20:4) | ≤ 0.353 | 100 | 45.83 | 0.708 | 0.1312 | 0.507 | 0.863 |
|  | PC(36:4) | ≤ -0.155 | 100 | 62.5 | 0.771 | 0.0030 | 0.574 | 0.907 |
|  | PC(38:6) | ≤ 0.147 | 100 | 58.33 | 0.760 | 0.0099 | 0.563 | 0.900 |
|  | Four lipids combined | -- | 100 | 79.17 | 0.875 | <0.0001 | 0.695 | 0.969 |
|  | PC(18:2_20:4)+ PC(36:4)+ PC(38:6) | -- | 100 | 66.67 | 0.792 | 0.0007 | 0.597 | 0.921 |
|  | CURB-65 | > 1 | 75 | 75 | 0.802 | 0.0057 | 0.609 | 0.927 |
|  | PSI | > 114 | 50 | 91.67 | 0.625 | 0.5246 | 0.423 | 0.799 |

**Abbreviations:** SCAP severe community-acquired pneumonia, CAP community-acquired pneumonia, NC non-CAP groups CURB-65 confusion, urea, respiratory rate, blood pressure, and age ≥65 years old score, PSI Pneumonia Severity Index score PC phosphatidylcholine
